# Supplementary material for: Determination of 14 Isoflavone Isomers in Natto by UPLC-ESI-MS/MS and Antioxidation and Antiglycation Profiles
Source: Foods. 2022 Jul 26;11(15):2229. doi: 10.3390/foods11152229 (PMC9332870; doi:10.3390/foods11152229)
Supplement: Supplementary file 1 [file foods-11-02229-s001.zip › foods-1814242-supplementary.pdf]

## 1. Single factor and orthogonal design experiment for extracting soybean isoflavones from natto

### *1.1. Single factor experiment of soybean isoflavone extraction*

Methanol is selected as the extractant because of the higher solubility of soybean isoflavones than in ethanol. On the yield of soybean isoflavones, the effects of methanol concentration, extraction time, extraction temperature, and liquid-solid ratio were investigated. Initially, the extraction efficiency of various aqueous methanol solutions was evaluated. Figure 2 (A) shows the total isoflavones extracted from 1g natto lyophilized powder using methanol aqueous solutions with various concentrations. Because of the viscous filamentous substances wrapped on the surface of natto, whose principal constituents are mainly polyfructose and polyglutamic acid, both of which are water soluble. Polyfructose and polyglutamic acid dissolve in water and wrap natto powder when the concentration of methanol aqueous solution is low (50%, 60%), which inhibits the dissolution of soybean isoflavones and reduces yield. When the concentration of methanol is high (90%), it is not conducive to the extraction of glycosidic soybean isoflavones with good water solubility. The greatest yield of soybean isoflavone extraction is 1.28 mg/g when the methanol concentration is 80%.

For the reasons stated above, we investigated the influence of the water bath time, extraction temperature as well as the liquid-solid ratio on isoflavone extraction yield. As shown in Figure 2 (B), the production of soybean isoflavones gradually increases as the extraction period is extended. The yield tends to be steady after the extraction period reaches 120 minutes. At this moment, the effect of extending the extraction time is not obvious. From the perspective of energy conservation, 120min is the best extraction time. It can be seen that a long enough water bath time allows the soybean isoflavones to completely dissolve. When the extraction time was 120 min, the yield of soybean isoflavones was 1.29 mg/g. The extraction efficiency of various water bath temperature was then assessed in Figure 2 (C). We used 30℃ to 70℃ extraction temperature for evaluating the yield of isoflavone from natto powder. It is found that when the temperature is below 50 ℃, the yield of soybean isoflavones increases as the temperature rises. When the temperature rises beyond 50℃, the yield of soybean isoflavones gradually declines. The reason for this could be that as the temperature rises, the thermally unstable soybean isoflavones disintegrate. As a result, the optimal extraction temperature is 50 °C, and the yield is 1.34 mg/g. The influence of different liquid-solid ratios on soybean isoflavone extraction efficiency was investigated as shown in Figure 2 (D), and it was discovered that when the material-liquid ratio is smaller than 30:1, soybean isoflavone yield increases with increasing of material-liquid ratio, and the difference is substantial. When the material-liquid ratio is greater than 30:1, the yield increase is not obvious. Therefore, 30:1 is chosen as the optimal extraction condition to save resources, and the yield is 1.21 mg/g.

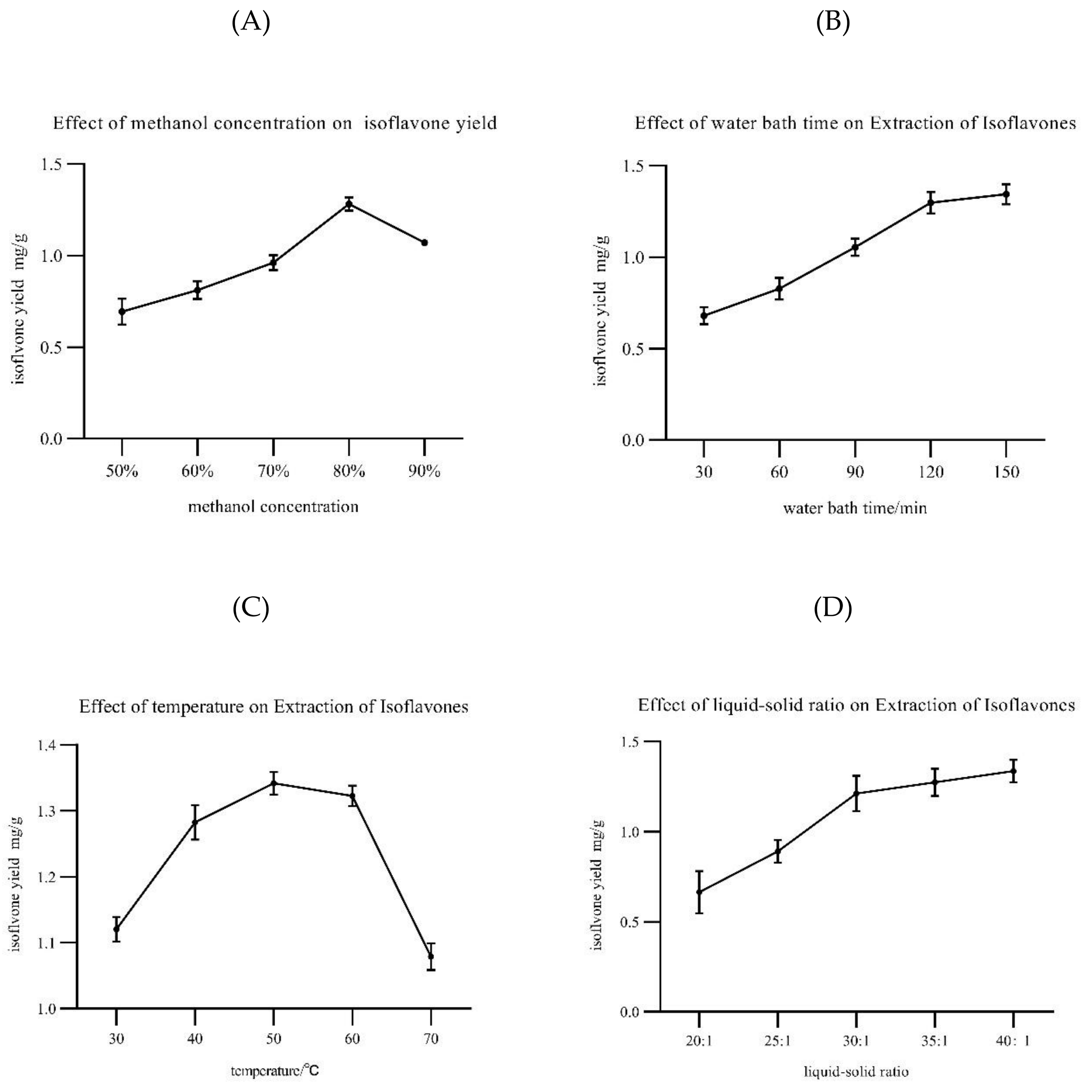


**Figure S1.** Effects of various factors on the yield of Soybean Isoflavones.

## *1.2. Orthogonal test*

The findings of the single factor experiment revealed that the following were the best extraction conditions for each factor: At a liquid-solid ratio of 30:1, an 80 percent methanol solution was extracted for 120 minutes at 50 °C. Methanol concentration (A), liquid material ratio (B), and water bath time (C), all of which have a significant impact on soybean isoflavone yield, were chosen to design a three factor and three-level orthogonal experiment based on the results of the single factor experiment.

The orthogonal experiment was designed with SPSS software, and the analysis of variance was performed, with the findings displayed in Table 2-1 and Table 2-2. Each factor was observed to have a significant influence on the extraction efficiency of soybean isoflavone in natto. The p-value was used to reflect the level of significance for each parameter. A value of p 0.05 is generally seen as indicating that the parameter has a significant effect. It can be seen from table 3 that all three factors had an impact on the yield of isoflavone extraction. The p-values of methanol concentration and liquid-solid ratio were 0.0003 and 0.006, respectively, indicating that these two factors had a significant effect, while the water bath time had little effect. In addition, the best scheme is $A_2B_3C_3$, and the yield of soybean isoflavones is improved to the greatest extent. The total soybean isoflavones in natto freeze-dried powder are extracted using optimized conditions, which

include a methanol concentration of 80%, a solid-liquid ratio of 1:35, and an extraction time of 150 minutes. The content of soybean isoflavones is 2.88±0.44 mg/ml, which is similar to the orthogonal experiment results.

**Table S1.** Effects of various factors on the yield of Soybean Isoflavones.

| Factor level | A methanol concentration (%) | B liquid-solid ratio | C water bath time (min) |
| --- | --- | --- | --- |
| 1 | 70% | 25：1 | 90 |
| 2 | 80% | 30：1 | 120 |
| 3 | 90% | 35：1 | 150 |

**Table S2.** Range analysis.

| Serial number | Level A | Level B | Level C | isoflavone yield |
| --- | --- | --- | --- | --- |
| 1 | 1 | 1 | 1 | 1.38 |
| 2 | 1 | 2 | 3 | 1.75 |
| 3 | 1 | 3 | 2 | 1.66 |
| 4 | 2 | 1 | 3 | 2.41 |
| 5 | 2 | 2 | 2 | 2.66 |
| 6 | 2 | 3 | 1 | 2.41 |
| 7 | 3 | 1 | 2 | 1.64 |
| 8 | 3 | 2 | 1 | 1.79 |
| 9 | 3 | 3 | 3 | 1.86 |
| Average 1 | 1.60 | 1.81 | 2.00 | |
| Average 2 | 2.63 | 1.79 | 1.99 | |
| Average 3 | 1.76 | 2.11 | 2.01 | |
| Range | 1.03 | 0.32 | 0.02 | |

**Table S3.** Analysis of variance.

| factor | Type III sum of squares | Degree of freedom | Mean square | F | P | Significance |
| --- | --- | --- | --- | --- | --- | --- |
| A | 2.088 | 2 | 1.044 | 2539.568 | 0.000 | *** |
| B | 0.138 | 2 | 0.069 | 168.189 | 0.006 | ** |
| C | 0.012 | 2 | 0.006 | 15.108 | 0.062 | * |
| Error | 0.001 | 2 | 0.000 | | | |

## 2. HPLC analysis of six isoflavone standards

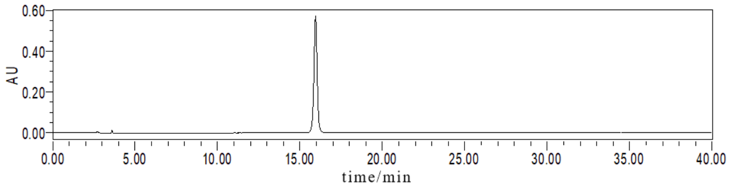


(1)

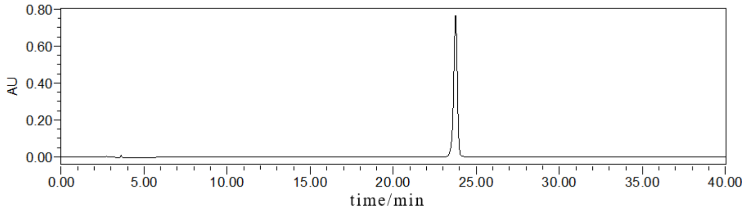


(2)

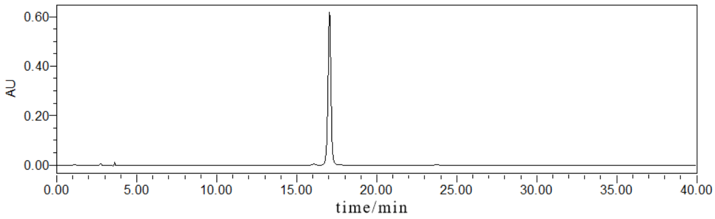


(3)

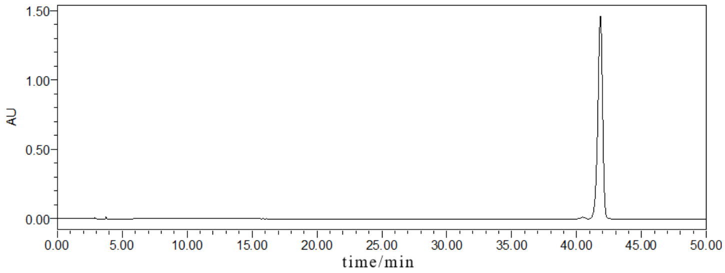


(4)

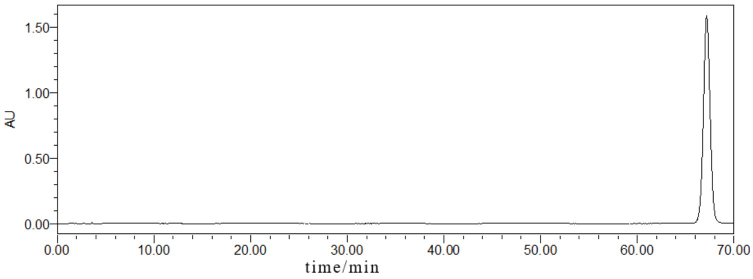


(5)

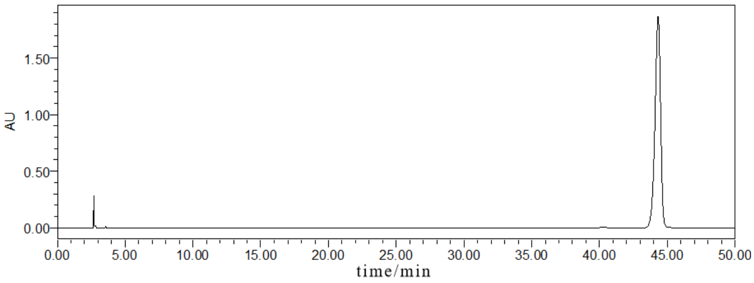


(6)

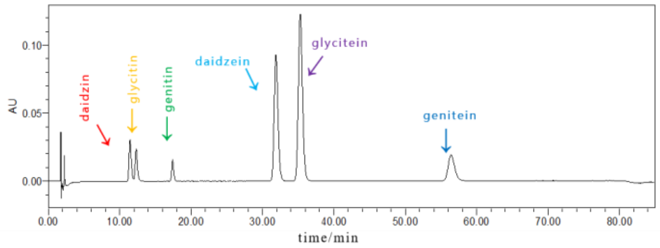


(7)

**Figure S2.** HPLC diagram of soybean isoflavone standard. Numbers of the diagrams are as follows: 1. daidzin standard, 2. Genistin standard, 3. Glycitin standard, 4. Daidzein standard, 5. Genistein standard, 6. Glycitein standard, 7. Mix Reference Material.

## 3. structure of soybean isoflavone isomers detected by UPLC-ESI-MS/MS

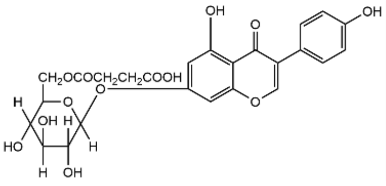

6''-O-succinyl-7-genistin

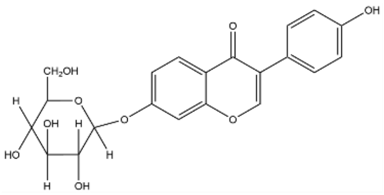

daidzin-7-glucoside

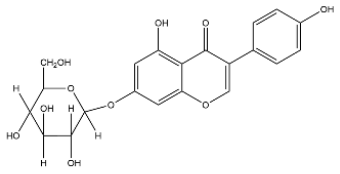

genistin-7-glucoside

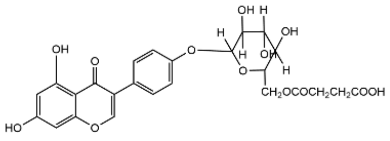

6''-O-succinyl-4'-genistin

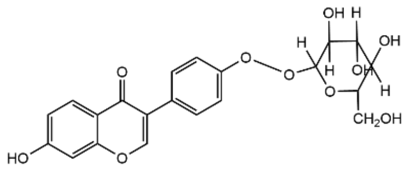

daidzin-4'-glucoside

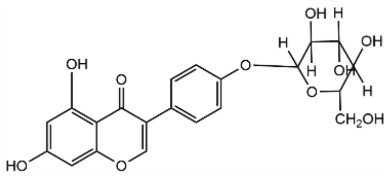

genistin-4'-glucoside

**Figure S3.** Soybean isoflavone isomers detected by UPLC-ESI-MS/MS Total ion flow.

## 4. diagram of isoflavones extracted from natto in positive ion mode

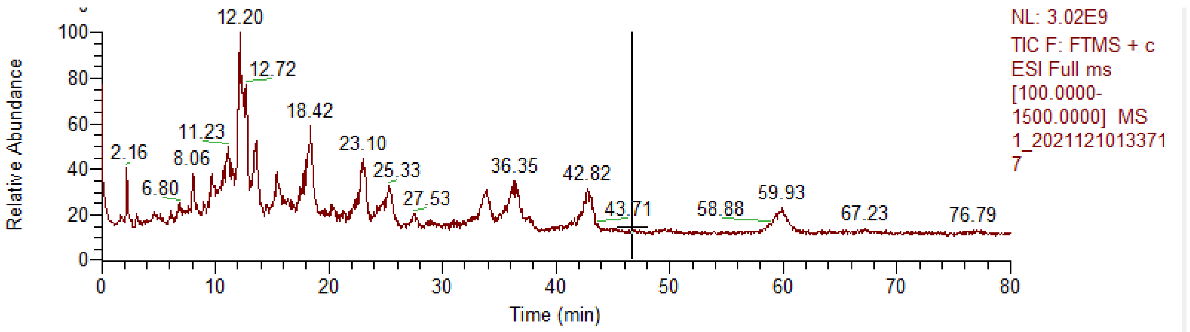


**Figure S4.** Total ion flow diagram of isoflavones extracted from natto in positive ion mode.
